# A Novel Recycling Route for Spent Li-Ion Batteries

**DOI:** 10.3390/ma15010044

**Published:** 2021-12-22

**Authors:** Eliana G. Pinna, Norman Toro, Sandra Gallegos, Mario H. Rodriguez

**Affiliations:** 1 Laboratorio de Metalurgia Extractiva y Síntesis de Materiales (MESiMat), ICB, UNCuyo, Consejo Nacional de Investigaciones Científicas y Técnicas, Facultad de Ciencias Exactas y Naturales, Padre Contreras 1300, Parque General San Martín, Mendoza CP M5502JMA, Argentina; mrodriguez@uncu.edu.ar; 2 Faculty of Engineering and Architecture, Universidad Arturo Prat, Iquique 1100000, Chile; ntoro@ucn.cl (N.T.); chichined@gmail.com (S.G.); 3 Cátedra de Química Analítica, Facultad de Ciencias Agrarias, Universidad Nacional de Cuyo, Almirante Brown 500, Chacras de Coria, Luján de Cuyo, Mendoza CPA M5528AHB, Argentina

**Keywords:** recycling, thermochemical, economical, LIBs

## Abstract

In this work, a recycling route for spent Li-ion batteries (LIBs) was developed. For this, the recovery of the metal content in both electrodes (anode and cathode) was investigated. Based on these results, an economic analysis of this recycling process was carried out. The obtained results showed that more than 90% of the material contained in both electrodes was recycled. The dissolution with acetic acid of the metals present in the active cathodic material is thermodynamically viable and the addition of a reducing agent such as hydrogen peroxide improved the spontaneity of the reaction. Dissolutions close to 100% for Li and Co were obtained. In addition, it was determined that the synthesis of lithium and cobalt valuable compounds was viable from the leach liquor, recovering approximately 90% of Co as cobalt oxalate, and 92% of Li as lithium carbonate. Furthermore, carbon graphite and Cu were fully recovered (100%) from the anodes. Finally, the results of the economic analysis showed that the recovered products have a high commercial value and industrial interest, providing an environmentally and economically viable process.

## 1. Introduction

Li-ion batteries (LIBs) are one of the most widely used energy accumulators today due to their energy advantages, such as a high charge density, high number of charge/discharge cycles, low self-discharge rate, and minimal maintenance, among others [1,2]. According to a report presented by Avicenne Energy Worldwide, the demand for LIB is expected to increase from 282 GW/h in 2020 to more than 2600 GW/h by 2030 [3].

LIBs are made up of two electrodes—an anode and a cathode. The anode is composed of graphite supported on a copper foil and the cathode contains, as an active material, an intercalated compound called mixed lithium oxide, LiMO_2_ (M: transition metals such as Co, Mn, and Ni, among the most studied), supported on an aluminum foil. The solids, graphite, and LiMO_2_ are attached to each sheet by fluoropolyvinyldiene (PVDF). Both electrodes are separated from each other by a porous plastic film, embedded in an electrolyte and covered by a metal and a plastic casing [2,3].

The average useful life of a LIB is 1.5 years, so the recycling of the contained metals, once they become disused, is very important, from both an environmental and economic point of view. Furthermore, the reuse of the metals contained in these devices would considerably reduce the manufacturing costs of new electrodes, without having to resort to obtaining them from natural sources through traditional mining. As a consequence of the current consumption trends, the global demand for key minerals for the manufacture of batteries is expected to increase significantly by 2028; only for lithium, it is expected that its demand will increase from 82 thousand metric tons (2020) to 1.9 million metric tons by 2028 [1,2,3,4,5].

The stages of sample preparation of the spent Li-ion batteries (LIBs) are very different, according to the different processes used: in some works, the authors have frozen these devices with liquid N_2_ (−197 °C), crushed them immediately after, and chemically treated the ground solid [6]. In others, LIBs are first completely discharged by immersing them in a NaCl solution and then they are manually opened, dismantled, and their parts are separated [6,7,8,9]. Subsequently, the solids adhered to the metal sheets (electrodes) can be separated using N-methyl-2-pyrrolidone (NMP), which dissolves the PVDF [10,11,12], or manually [6,7,8,9]. Then, the powder or paste is generally washed and a heat treatment is performed, between 400–900 °C and for 1 to 5 h, in order to remove the remains of solvents and/or adhesive [2,13].

The hydrometallurgical process consists of acid leaching of the metallic content in the LIBs and their recovery through different pathways. This methodology offers advantages such as low energy consumption, minimum release of toxic gases, short reaction times, and recovery of high purity products [2,14].

According to Huang et al. [15], the LCO structure is one of the most commonly used for the manufacture of Li-ion batteries, giving place to a large amount of work aimed at studying the extraction of the metals that compose it [13]. Many inorganic acids have been used, with and without H_2_O_2_, such as HNO_3_ [16,17], H_2_SO_4_ [18,19], H_3_PO_4_ [6,20,21], HF [7,9], and HCl [19,22] for the dissolution of the metals contained in LCO due to the high efficiency obtained, between 60–90%. However, given that they can emit toxic gases (Cl_2_, F_2_, SO_3_, and NO_x_), corrode the equipment and generate liquid effluents of expensive treatment, in recent years, carboxylic acids have started to be studied as an alternative to inorganic agents. Most carboxylic acids have less acidity than inorganic acids, but compensate for this deficiency with the chelating effect they possess, favoring the extraction of these metals. This benefit is due to the fact that the hydrogen cations attack the metals and mobilize them into the solution. The organic compounds then stabilize the metal ions by forming a complex or chelate. In addition, carboxylic acids are generally biodegradable, thermally stable, do not corrode the equipment, and the emission of toxic gases is minimal [23,24].

The works reported in the bibliography, in which carboxylic acids are used to dissolve the LCO-type cathodic material in the presence of a reducing agent such as H_2_O_2_, report the use of the acids malic [25,26], citric [10,25,26,27,28], aspartic [25], ascorbic [29], lactic [30], gluconic [30], formic [31], and succinic [32]. According to the results, citric acid generates the best dissolutions: 100% of LCO. Other investigations studied the dissolution of LCO using ascorbic acid as a reducing agent and the following acids as chelating agents: citric [33], tartaric [34], iminodiacetic [35], malic [35], nitrilotriacetic [36], and adipic [36].

For all the above, there is an important need to study the complete recycling process for LIB electrodes. That is why, in this work, the composition and the methods of recovery of the elements contained in both electrodes were first studied. Then, the operational parameters of the LCO (LIBs cathode active material) dissolution process were investigated using acetic acid and hydrogen peroxide as the leaching and reducing agents, respectively. Later, the recovery of Li and Co was carried out from the leaching liquors. Finally, an economic analysis of the studied process was carried out to determine its viability.

## 2. Materials and Methods

### 2.1. Materials and Chemical Reagents

The LIBs used in this study were provided by the district of Godoy Cruz, Mendoza, Argentina. These batteries came from disused mobile phones.

The chemical reagents used were glacial acetic acid (Alkemit, Argentina, ACS, 99.7%), oxalic acid (Biopack, Argentina, ACS, 99.8%), carbon dioxide compressed gaseous (Praxair, Argentina, 99.0%), and hydrogen peroxide (Biopack, Argentina, ACS, 30% *w/v*). Solutions with calculated volumes of deionized water were prepared. 

### 2.2. Dismantling of the Batteries

**Figure 1:**
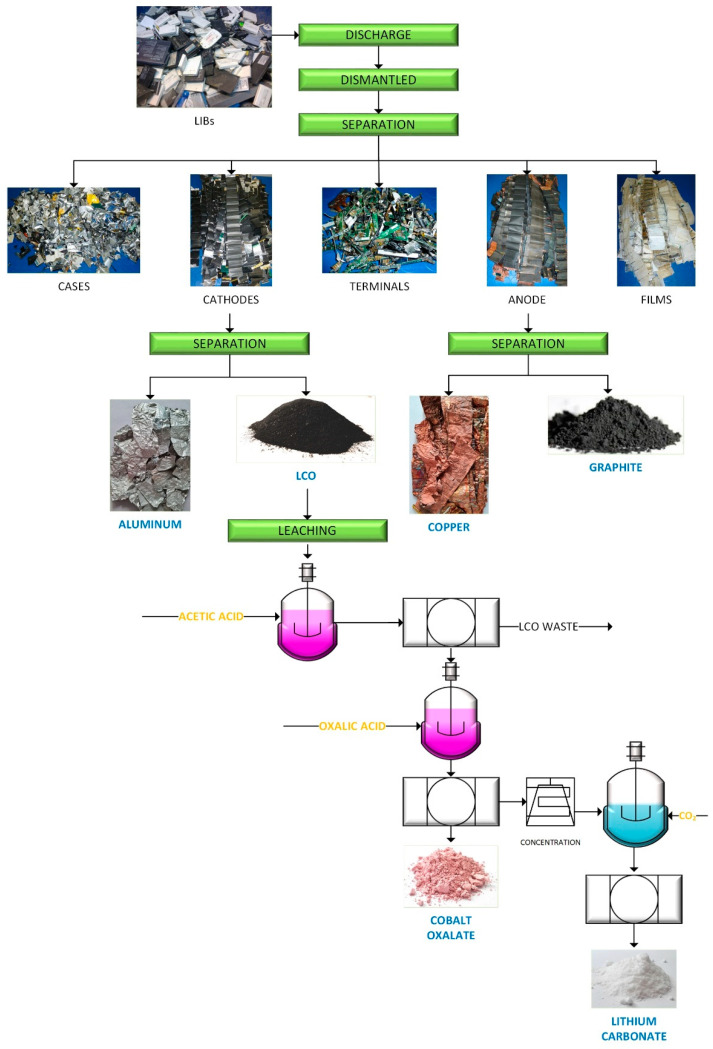
Process flow diagram.

The sample was prepared using 1000 LIBs of mobile phones from different brands. Figure 1 presents a diagram of the complete recovery process. The dismantling and obtaining of the samples for the leaching tests can be seen.

**Figure 2:**
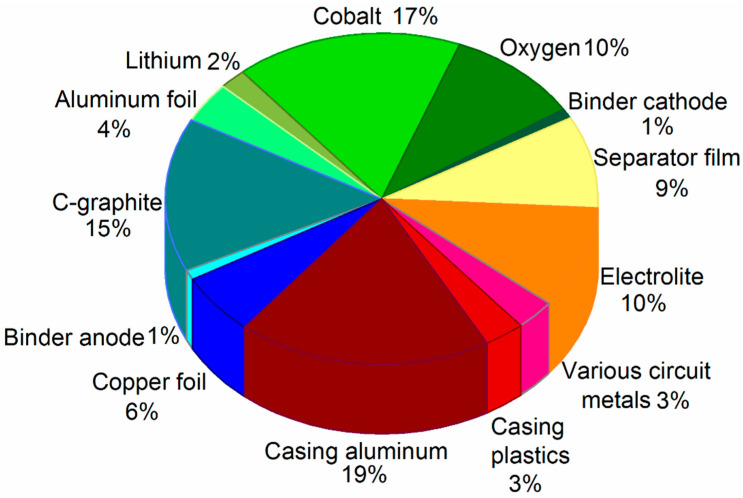
Composition % weight of LIBs.

In Figure 2, the percentage by weight of the different parts that were separated from the LIBs are presented. It can be seen that 34% corresponds to the cathode, 22% to the anode, 22% to the casing, 9% to the separator film, 10% to the electrolyte, and 3% to the circuit and terminals. Of all the content, we focused on studying the recycling of the anode, cathode, and separating film (65% by weight). In future studies, we will present the recycling of the housing, terminals, circuit, and electrolyte.

### 2.3. Leaching Procedure

The dissolution tests of the active material of the cathodes were conducted in a closed batch reactor. This reactor had a temperature and stirring control system.

In each test, the mass of the active cathode material and the volume of deionized water required was calculated. These were placed inside the reactor and were heated with stirring until reaching the working temperature. At that moment, the calculated volumes of the leaching agent and the reducing agent were added and the reaction time began to be measured.

In this work, a univariate analysis of the experimental leaching conditions was performed. Studied variables and intervals were as follows: concentration of acetic acid (0.3–2.8 M), concentration of H_2_O_2_ (0–10% *v/v*), temperature (15–90 °C), reaction time (15–300 min), stirring speed (0–440 rpm), and a solid−liquid ratio (1–20 g/L). The tests were performed in triplicate to avoid random errors.

The percentages of dissolved metals were calculated using the following expression [8]:

(1) $$Metal\;dissolution\;\%=\frac{m^{d}}{m^{0}}\times 100$$

where *metal* (lithium or cobalt) *dissolution* % is the percent dissolution efficiency, *m*^0^ is the initial metal concentration in the sample, and *m^d^* is the dissolved metal of the sample. 

### 2.4. Precipitation Procedure

The metals dissolved during the leaching (lithium and cobalt) were recovered by chemical precipitation. Cobalt was first recovered, as cobalt oxalate, using oxalic acid as a precipitating agent. Once the cobalt salt had precipitated, it was washed several times to remove impurities and was dried at 50 °C. Later, the obtained liquor was pre-concentrated by evaporation until obtaining a concentration of 10 g/L of lithium. Next, carbon dioxide was bubbled in to precipitate lithium as the carbonate. The precipitate obtained was washed several times to remove impurities and was dried at 50 °C [7].

The recovered metals were analyzed by AAS in all of the tests. The recovery percentage was calculated by the following:

(2) $$Metal\;recovery\;\%=\frac{E^{i}}{E^{f}}\times 100$$

where *E^i^* is the initial amount of each element in the sample and *E^f^* is the quantity of each element in the leach liquor obtained after precipitation.

### 2.5. Analytical Methods

The analytical techniques used for the characterization were scanning electron microscopy (SEM) using a LEO 1450 VP microscope with an EDAX Genesis 2000 (EDS) X-ray dispersive spectrometer (Zeiss, Jena, Germany), X-ray diffractometry (XRD) with a Rigaku D-Max III C diffractometer (Rigaku, Osaka, Japan), atomic absorption spectroscopy (AAS) with a Varian SpectrAA 55 spectrometer (Palo Alto, CA, USA), and X-ray fluorescence spectroscopy (XRF) with a Shimadzu EDX 7000 (Shimadzu, Kyoto, Japan).

## 3. Results and Discussion

### 3.1. Recycling of the Anode

The anodes of the spent Li-ion batteries were treated with de-ionized water and magnetic stirring at 50 °C to separate the copper sheets from the carbon graphite. Here, 5.2 kg of active material from the anode and 0.8 kg of copper were obtained from the batteries under study. The active material from the anode, after being separated, was washed and dried at 400 °C to completely remove the PVDF. 

**Figure 3:**
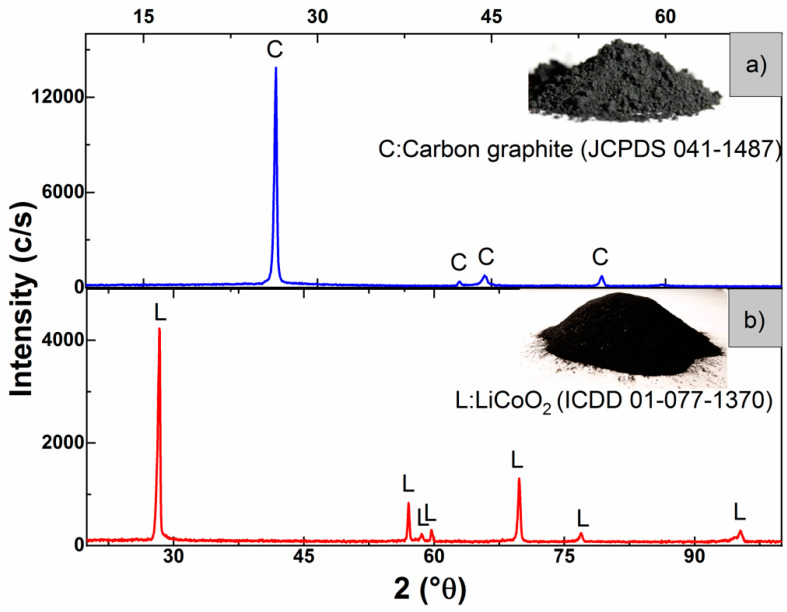
Results of the characterization of the active material of the (**a**) anode and (**b**) cathode, by XRD.

Figure 3a presents the characterization by XRD of the obtained powder. It can be seen that the diffraction lines of the recovered material corresponded to C-graphite (JCPDS 041-1487). Furthermore, Table 1 presents the chemical analysis of the elemental composition, carried out by AAS and XRF, of the active materials recovered from the anodes. It can be seen that the recovered material was mostly C-graphite.

### 3.2. Recycling of the Cathode

The cathodes extracted from the spent Li-ion batteries under study were subjected to a mechanical treatment in which the aluminum sheets (1.1 kg) and the mixed lithium oxide (7.75 kg) were separated. The recovered active material was washed, calcined at 400 °C (to remove adhered glue), and mixed in a mill to obtain a homogeneous and representative sample.

Next, we studied a process of separation and recovery of the metals contained in the active material recovered from the cathodes. In the diffractogram of Figure 3b, the presence of a crystalline structure corresponding to the oxide of lithium and cobalt (ICDD 01-077-1370) in the active material of the cathode is observed. In addition, in Table 1, it can be seen that the composition of the obtained mixed oxide is mostly represented by lithium and cobalt.

#### 3.2.1. Thermochemical Analysis of the Leaching of Waste LCO 

The thermodynamic analysis was performed using HSC 5.1 Software. Table 2 shows the equations proposed for the dissolution of LCO with acetic acid, with and without the presence of a reducing agent, and their values of free energy of Gibbs calculated at different temperatures. 

Table 2 shows that the ΔG values were negative, which would indicate that both of the proposed reactions were spontaneous in the temperature ranges studied. Furthermore, from the values of ΔG, it is observed that the dissolution reaction was thermodynamically favored by adding a reducing agent such as hydrogen peroxide. Therefore, below, we focused on analyzing and investigating the best leaching conditions for the separation of Co and Li from LCO with acetic acid.

#### 3.2.2. Analysis of the Dissolution of the LCO

##### Effect of Solid/Liquid Ratio

**Figure 4:**
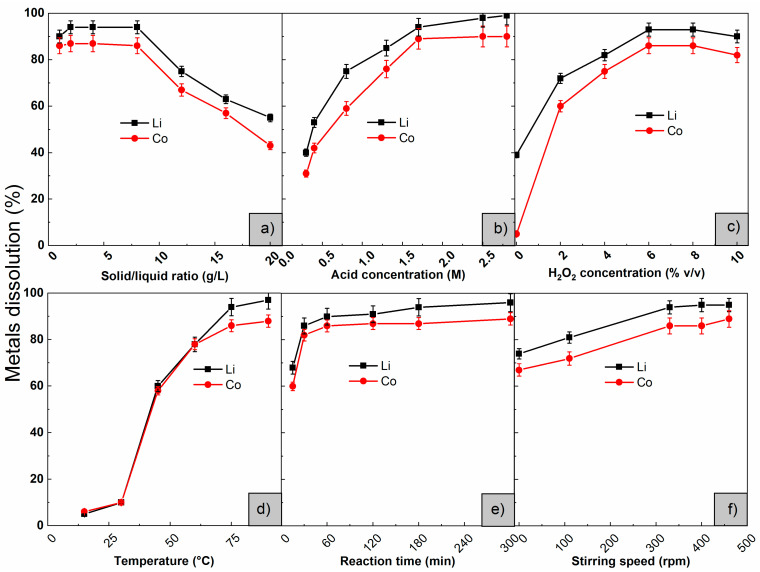
Effect of (**a**) solid/liquid ratio, (**b**) acid concentration, (**c**) H_2_O_2_ concentration, (**d**) temperature, (**e**) reaction time and (**f**) stirring speed.

The effect of the solid−liquid ratio on the leaching of Li and Co was studied between 1–20 g/L. The conditions for the leaching reaction were as follows: temperature of 75 °C, stirring speed of 330 rpm, and reaction time of 60 min. The results (Figure 4a) show that a solid−liquid ratio between 1–8 g/L has a low influence on the dissolution reaction of the metals. However, when increasing the solid−liquid ratio above 8 g/L, there was a decrease in the dissolution of the sample, which may be due to the lower availability of ions reacting with the mixed oxide and forming soluble compounds.

##### Effect of Leaching Agent Concentration

The studied operational parameters were temperature of 75 °C, time of 60 min, stirring speed of 330 rpm, S/L ratio of 8 g/L, and concentration of H_2_O_2_ of 2% *v/v*. The results shown in Figure 4b indicate that an increase in the concentration of the leaching agent has a marked influence on the leaching of both metals. The best dissolutions were obtained from 1.7 M. This supposes a significant benefit in comparison to the use of conventional inorganic leaching agents, such as, HCl, H_2_SO_4_, and HNO_3_, which, in order to reach these dissolution values, required concentrations of 2 to 4 M [2].

##### Effect of H_2_O_2_ Concentration

The studied operational parameters were temperature of 75 °C, time of 60 min, stirring speed of 330 rpm, S/L ratio of 8 g/L, and concentration of HAc of 1.7 M. Figure 4c shows that the increase in the concentration of H_2_O_2_ up to 6% *v/v* favored the dissolution of Li and Co from LCO. This was verified in the calculated Gibbs free energy values, as the reaction was thermodynamically favored by the addition of hydrogen peroxide. Besides, when H_2_O_2_ was added to the carboxylic acid-LCO system, increases in the dissolution higher than 30% for Co and 10% for Li were observed. According to Li et al. [32], the bond Co-O of LCO was extremely strong, thus its dissolution with a weak acid was difficult. When H_2_O_2_ was added to the reacting system, the oxygen generated by the decomposition of H_2_O_2_ reduced Co (III) to Co (II), which increased the dissolution [6]. Nayaka et al. [33] presumed that the increase in dissolution was due to the mechanism of formation of reducing complexes. Co^3+^ (d^6^, r = 0.63 Å) in the reticulum of the oxide exited easily by reduction to Co^2+^ (d^7^, r = 0.74 Å), because the difference in the ionic radius destabilized the crystalline structure of the solid. Then, the ion was stabilized by complexation with a chelating agent. 

When the concentration of H_2_O_2_ was above 6% *v/v*, a diminution in the dissolution of the sample occurred. When an excess of hydrogen peroxide was added, its reducing function changed to oxidizing, which would explain the decrease in the extraction of the metals.

##### Effect of Temperature

The assays were carried out using concentrations of the leaching agent of 1.7 M, H_2_O_2_ 6% *v/v*, 60 min, 330 rpm, and S/L ratio of 8 g/L. The increase in the temperature (Figure 4d) promoted the leaching reaction. This happened in the first place because the reaction of dissolution of metals is an endothermic process [37] and in the second place because the average kinetic energy of the molecules increases along with the temperature, which causes more frequent and energetic collisions, leading to an increase in dissolution. At temperatures above 80 °C, the dissolution remained constant because H_2_O_2_ began to decompose.

##### Effect of Reaction Time

The experiments were performed using concentrations of HAc of 1.7 M, H_2_O_2_ 6% *v/v*, 75 °C, 330 rpm, and S/L ratio of 8 g/L. The results presented in Figure 4e indicate that an increase in reaction time increased the extractions of both Li and Co because the time of contact among reactants favored the dissolution reaction. Best leaching results (close to 100%) were reached after 60 min. It is worth mentioning that for 30 min, high dissolutions of LCO were achieved, higher than 80%, which is beneficial if the process is to be escalated.

##### Effect of Stirring Speed

The effect of stirring speed on the cathode active material dissolution was investigated from 0 to 500 rpm. The experiments were performed using concentrations of HAc of 1.7 M, H_2_O_2_ 6% *v/v*, 75 °C, 60 min, and an S/L ratio of 8 g/L. The results shown in Figure 4f indicate that a variation in the stirring speed between 0–500 rpm did not affect the extraction of the metals. This would indicate that the rate-determining step was the chemical reaction.

#### 3.2.3. Characterization of the Solid Residues

**Figure 5:**
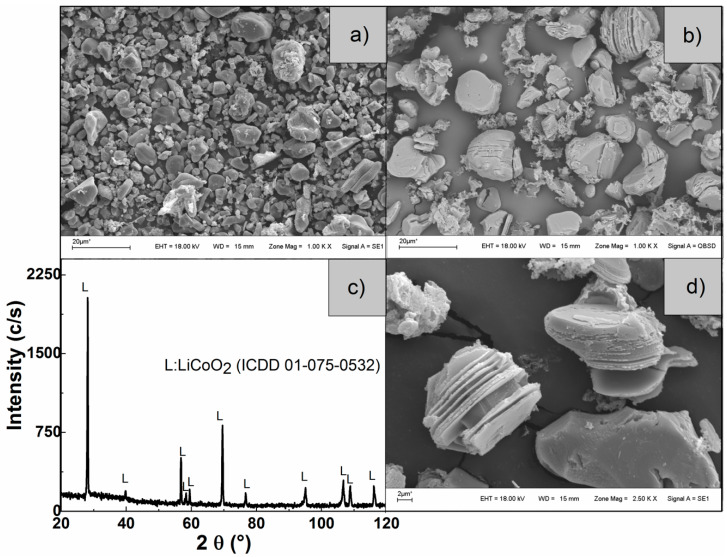
SEM micrographs of (**a**) active material cathode, (**b**,**d**) of the leaching residues obtained, and (**c**) diffractogram of the leaching residue obtained.

Figure 5 shows the characterization by XRD and SEM of the solid residues of the sample leached with acetic acid at 75 °C, 60 min, 330 rpm, S/L ratio of 8 g/L, concentrations of the leaching agent of 1.7 M, H_2_O_2_ 6% *v/v*.

The micrographs of Figure 5b,d show a change in the morphology of the leaching residue with respect to the sample (Figure 5a). The particles of the residues obtained with HAc show attacked particles, but partially preserving their size and shape. Figure 5c shows the diffractogram of the residue where only diffraction lines corresponding to undissolved LiCoO_2_ (ICCD 01-075-0532) were detected, which enabled us to deduce that no crystalline solids were formed.

#### 3.2.4. Recovery of Metals

The recovery of lithium and cobalt in the solution was carried out in two steps. Cobalt oxalate was first obtained (step 1) by precipitation with oxalic acid, according to Equation (3). Then, in a second step, the previously filtered liquid was used, it was pre-concentrated by evaporation at 80 °C and CO_2_ was bubbled until reaching pH 13 to obtain Li_2_CO_3_, according to Equation (4). The recovery percentage of both metals followed Equation (2). In step 1, 99% of cobalt was recovered. In the second stage, a recovery of 92% of Li was obtained

Co^2+^_(aq)_ + H_2_C_2_O_4_ → Co[C_2_O_4_]_(s)_ + 2H^+^_(aq)_ (3)

2Li^+^_(aq)_ + H_2_O + CO_2(g)_ → Li_2_CO_3_ + 2H^+^_(aq)_ (4)

**Figure 6:**
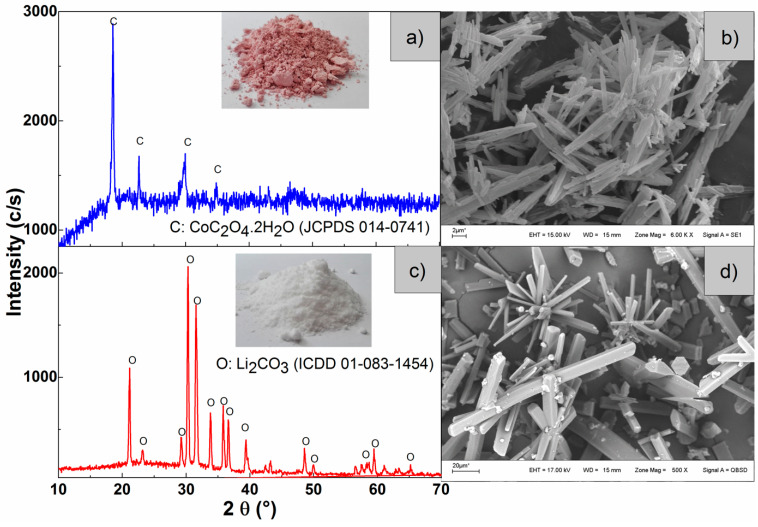
Diffractograms and micrographs of the synthesized solids. (**a**) XRD and (**b**) SEM of the cobalt oxalate, (**c**) XRD, and (**d**) SEM of the lithium carbonate.

The solids obtained in both stages were washed, dried, and then characterized by XRD, SEM, and EPMA. In Figure 6a, it can be seen that the diffraction lines of the solid precipitated in step 1 correspond to cobaltous oxalate dihydrate (JCPDS 014-0741) whose crystals are rod-shaped with a diameter of between 2 and 15 µm, as can be seen in Figure 6b. Figure 6c shows that the precipitate formed in step 2 is Li_2_CO_3_ (ICDD 01-083-1454). These synthesized crystals, as shown in Figure 6d, have a morphology corresponding to a monoclinic crystal structure.

In Table 3, the results of the EPMA analysis of the synthesized solids shown in Figure 6b,d are presented. It can be seen that both cobalt oxalate and lithium carbonate had excellent purity levels of 98.3 and 99.6%, respectively. The Mn detected in the cobalt oxalate came from the original sample and would not interfere with its potential applications.

### 3.3. Preliminary Economic Study

**Figure 7:**
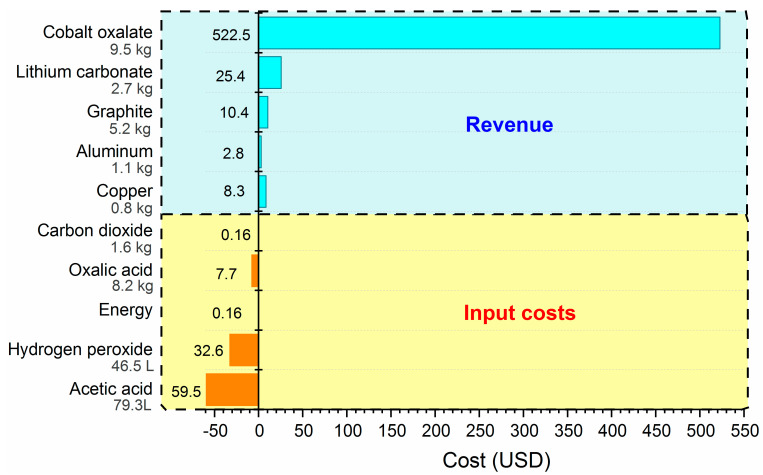
Costs of the recycling process of 1000 cellphone LIB modules.

The preliminary economic analysis was carried out without considering the costs of collection, transportation, assembly, and operation of the plant. Here, only the manufacturing costs of the process were analyzed. It is important to note that the costs of the complete process varied considerably depending on the geographic location of a plant. That is why, in this work, only the manufacturing costs of the process proposed in Figure 1 were analyzed. In Figure 1, the incoming reagents of the system are shown in yellow and the compounds recovered from the LIBs are in blue. The prices of the chemical products used and obtained in the recycling process were obtained from the bibliography [38,39]. The electrical cost was taken as $0.09 per kWh [38,39]. The results in Figure 7 show the cost of the recycling process of 1000 cells of LIBs from mobile phones. In addition to the cost (input cost), calculating the cost of recycling LIBs also considered revenues, which were calculated as follows:

(5) $$Revenue=\sum^{\;}m_{i}\times up_{i}$$

where *m_i_* is the mass of material that was recovered from LIBs and *up_i_* is the unit price of the material. 

For the calculations, the dissolution and recovery percentages studied in Section 3.2.2 and Section 3.2.4 for the active cathode material were taken into account. In addition, the purities of the obtained products were also taken into account.

Figure 7 shows us that the complete LIB recycling process is economically viable mainly because it obtains products with high purities and good economic prices. In this preliminary economic study, it was calculated that from the analyzed package to be recycled, the following would be obtained: 1.1 kg of aluminum, 0.8 kg of copper, 5.2 kg of graphite, 2.7 kg of lithium carbonate, and 9.4 kg of cobalt oxalate.

## 4. Conclusions

A route to almost completely recycle the content of the material of LIBs was developed, in particular, 90% mass contents from both electrodes. C-graphite and copper, from the anode, and aluminum and the cathodic active material (LCO) from the cathodes were recovered. Then, LCO dissolutions close to 100% were obtained, extracting almost all the Li and Co, with acetic acid in a reducing medium. Finally, about 90% of the Co (as cobalt oxalate) and Li (as lithium carbonate) were recovered. The salts of the recovered metals had high purities, above 98%. The preliminary economic analysis showed that the recovered products were of a high commercial value, leading to the viability of the proposed route.

## Figures and Tables

**Table 1:** Atomic percentage of the electrode active materials composition.

Elements (%)	Li	Co	Mn	Ni	C	Al	Fe	Cu
Cathode active materials	7.34	54.56	2.07	1.78	1.02	0.29	0.14	---
Anode active materials	1.70	-----	-----	-----	97.30	-----	-----	1.00

**Table 2:** Proposed LCO dissolution equations.

Proposed Equations	ΔG			
	298 K	323 K	348 K	363 K
4LiCoO_2_ + 12CH_3_COOH + 2H_2_O_2_ = 4Li^+^ + 4Co^2+^ + 8H_2_O + 2O_2(g)_ + 12CH_3_COO^−^	−109.3	−102.9	−95.4	−90.3
4LiCoO_2_ + 12CH_3_COOH = 4Li^+^ + 4Co^2+^ + 6H_2_O + O_2(g)_ + 12CH_3_COO^−^	−60.0	−53.2	−45.3	−39.9

**Table 3:** EPMA results of analysis of the synthesized solids, as shown in Figure 6b,d.

Compounds	Purity	% Atomic					
		Mn	Ni	Fe	Co	C	O
CoC_2_O_4_	98.3%	1.7	--	--	31.08	31.40	35.81
Li_2_CO_3_	99.6%	--	--	--	--	18.21	62.41

## Data Availability

Not applicable.
